# RETRACTED: Ikram et al. Cycloastragenol, a Triterpenoid Saponin, Regulates Oxidative Stress, Neurotrophic Dysfunctions, Neuroinflammation and Apoptotic Cell Death in Neurodegenerative Conditions. *Cells* 2021, *10*, 2719

**DOI:** 10.3390/cells15030257

**Published:** 2026-01-29

**Authors:** Muhammad Ikram, Myeung Hoon Jo, Kyonghwan Choe, Amjad Khan, Sareer Ahmad, Kamran Saeed, Min Woo Kim, Myeong Ok Kim

**Affiliations:** 1 Division of Life Science and Applied Life Science (BK21 Four), College of Natural Sciences, Gyeongsang National University, Jinju 52828, Republic of Korea; qazafi417@gmail.com (M.I.); audgns1217@gnu.ac.kr (M.H.J.); amjadkhan@gnu.ac.kr (A.K.); sareer_50@gnu.ac.kr (S.A.); kamran.biochem@gnu.ac.kr (K.S.); mwkim0322@gnu.ac.kr (M.W.K.); 2 Department of Psychiatry and Neuropsychology, School for Mental Health and Neuroscience (MHeNs), Maastricht University, 6211 LK Maastricht, The Netherlands; k.choe@maastrichtuniversity.nl; 3 Alz-Dementia Korea Co., Jinju 52828, Republic of Korea

The journal retracts the article titled “Cycloastragenol, a Triterpenoid Saponin, Regulates Oxidative Stress, Neurotrophic Dysfunctions, Neuroinflammation and Apoptotic Cell Death in Neurodegenerative Conditions” [1], cited above.

Following publication, concerns were brought to the attention of the Editorial Office regarding the integrity of several images in this publication [1].

In accordance with standard journal procedures, the Editorial Office and Editorial Board conducted an investigation that confirmed inappropriate editing, partial overlap in Figures 2, 3 and 4 and partial duplication in Figures 2 and 3 of this publication [1] with content from earlier publications [2,3,4] by a similar group of authors presenting different experimental conditions. While the authors cooperated during the investigation, they were unable to satisfactorily explain the duplication or provide the original images that met the Editorial Board’s requirement.

As a result, the Editorial Board has lost confidence in the reliability of the findings and has decided to retract this publication, as per MDPI’s retraction policy (https://www.mdpi.com/ethics#_bookmark30).

This retraction was approved by the Editor-in-Chief of the journal *Cells*.

The authors have been informed of this retraction and have indicated their disagreement with this decision.
